# LncRNA SNHG17 aggravated prostate cancer progression through regulating its homolog SNORA71B via a positive feedback loop

**DOI:** 10.1038/s41419-020-2569-y

**Published:** 2020-05-23

**Authors:** Gaoliang Wu, Chao Hao, Xueliang Qi, Jianqiang Nie, Weimin Zhou, Ji Huang, Qiuming He

**Affiliations:** Urology, Jiangxi cancer hospital, Nanchang, 330029 Jiangxi China

**Keywords:** Prostate cancer, Cell biology

## Abstract

Prostate cancer (PC) is a prevalent male malignancy with high occurrence rate. Recent studies have showed that small nucleolar host genes (SNHGs) and their homolog small nucleolar RNAs (snoRNAs) elicit regulatory functions in carcinogenesis. Present study aimed to investigate the role of SNHG17 and its homolog SNORA71B in PC. Function of SNHG17 and SNORA71B in PC is detected by CCK-8, colony formation, flow cytometry analysis of apoptosis, and transwell migration assay. The mechanism whereby SNHG17 regulated SNORA71B was detected by RIP, pulldown, ChIP, and luciferase reporter assays. Results depicted that transcript 6 of SNHG17 and SNORA71B were upregulated in PC. Knockdown of SNHG17 or SNORA71B weakened proliferation, invasion, migration, and epithelial-to-mesenchymal transition (EMT) and strengthened apoptosis. Mechanistically, SNHG17 and SNORA71B were transcriptionally activated by signal transducer and activator of transcription 5A (STAT5A). SNHG17 positively regulated SNORA71B in PC cell lines and other cell lines. SNHG17 sponged miR-339-5p to upregulate STAT5A and therefore to cause transactivation of SNORA71B. Rescue experiments delineated that SNORA71B was required for the regulation of SNHG17 on PC. Moreover, SNHG17 silence hindered tumorigenesis of PC in vivo. In conclusion, current study first revealed that lncRNA SNHG17 aggravated prostate cancer progression through regulating its homolog SNORA71B via a positive feedback loop, which might do help to the pursuit of better PC treatment.

## Introduction

Prostate cancer (PC) ranks the second among the global common malignancies and is the major contributor of the mortality of male cancer worldwide^1^. Generally, race, age, and heredity are considered as the major risk factors of PC. Recent findings show that alternative splicing of mRNA, methylation of DNA, and dysregulation of steroidogenic enzyme caused by androgen stimulation are also important factors for PC development^2^. Therefore, better understanding of the detailed molecular mechanism in PC pathogenesis may improve the efficacy of clinical treatment^3^.

Long non-coding RNAs (lncRNAs) belong to non-coding RNAs (ncRNAs) whose length is over 200 bp^4^. Volumes of works have stated that lncRNAs are functional in carcinogenesis, regulating proliferation, invasion, EMT, apoptosis and so on^5–7^. Also, the link between lncRNAs and PC progression is well established^8–10^. Small nucleolar host genes (SNHGs) are a subset of long RNAs which are spliced from the exons of the primary RNA transcripts of the SNHGs and transported to the cytoplasm of cells. Multiple SNHGs have been reported to participate in cancer progression, including PC^10–13^. Interestingly, besides SNHGs, small nucleolar RNAs (snoRNAs) are another group of ncRNAs that are spliced from introns of the primary transcripts of SNHGs^14^, mainly expressed in nucleus and functioning as the guiders of posttranscriptional modification of small RNAs^15^. Also, snoRNAs are discovered to show deep implication with the development of diverse malignancies^14^. These findings suggested the potential affinity between SNHG-transcribed lncRNAs and snoRNAs. SNHG17 has been reported to elicit oncogenic influences in gastric cancer and colorectal cancer^16,17^. However, the relation between SNHG17 and PC remains unexplored. In addition, small nucleolar RNA 71B (SNORA71B), the homolog of SNHG17, is demonstrated to be upregulated in breast cancer^18^, but never has it been associated with PC and SNHG17 before.

MicroRNAs (miRNAs) are a sub-class of small ncRNAs consisting of approximately 22 nucleotides^19^. They have been characterized as essential administrators of gene expression, considering that miRNAs can complementarily bind to the target mRNAs to result in mRNA degradation and prevent the production of functional proteins^20^. To date, miRNAs are widely investigated in tumorigenesis and cancer progression^21–23^, including in PC^24–26^. Furthermore, numerous reports explained the competitive endogenous RNA network mediated by lncRNA-miRNA-mRNA interaction in cancer progression, through which the lncRNAs act as miRNA sponge and induce gene expression in cancer cells^27–29^. In PC, the ceRNA module is largely revealed as well^10^. MiR-339-5p has been recognized as a tumor-suppressor miRNA in myriads of cancers such as ovarian cancer^30^, lung adenocarcinoma^31^, breast cancer^32^, and colorectal cancer^33^. However, there is no report about the involvement of miR-339-3p in PC and its interaction with SNHG17.

Current study planned to investigate the function of SNHG17 and SNORA71B in PC and the regulatory mechanism of SNHG17 on SNORA71B.

## Materials and methods

### Microarray analysis

Total cellular RNAs were extracted by use of TRIzol reagent from Thermo Fisher Scientific (Waltham, MA, USA). Whole-genome microarray expression profiling was conducted with a cut-off criteria of *p* < 0.05 and log2 (fold change) > 2 for screening out the differentially expressed genes.

### Human tissue samples

Thirty-six PC specimens and adjacent non-tumor tissues were gained from patients who did not receive any therapy before undergoing operation in Jiangxi cancer hospital. Tissue specimens were obtained from patients who signed informed consent. Approval of the study protocol was obtained from the Jiangxi cancer hospital. All methods of this experiment were rigidly performed in line with the approved guides. Immediately after the operation, tissue samples were frozen in liquid nitrogen and kept at −80 °C.

### Cell culture

Normal human prostate epithelial cell (RWPE-1), PC cells (DU145, LNCaP, VCaP, PC-3), non-small cell lung cancer (NSCLC) cell line A549, colorectal cancer cell line DLD-1, and human renal epithelial cell (239T) were all obtained from American Type Culture Collection (ATCC; Manassas, VA, USA). Gastric cancer cell line MGC-803 was bought form Cell Bank of the Chinese Academy of Sciences (Shanghai, China). Cells were maintained in DMEM (Gibco, Waltham, MA, USA) supplemented with 10% fetal bovine serum (FBS; Gibco), 100 U/mL penicillin and streptomycin (Gibco) at 37 °C with 5% CO_2_.

### Cell transfection

The short hairpin RNAs (shRNAs) for SNHG17 (sh-SNHG17#1/2/3), STAT5A (sh-STAT5A#1/2/3) and their negative controls (shNCs) were obtained from Keygen Biotech (Nanjing, Jiangsu, China), along with the antisense oligonucleotides (ASOs) for inhibiting SNORA71B. MiR-339-5p mimic and miR-339-5p inhibitor, as well as NC mimic and NC inhibitor were constructed by Keygen Biotech. Overexpression of SNORA71B cell models were established by lentivirus (Lv-SNORA71B) (GeneCopoecia, Guangzhou, China). Cells in 6-well plates at a density of 80–90% were transfected with Lipotransfectamine 3000 (Thermo Fisher Scientific) for 48 h.

### Quantitative real-time PCR (RT-qPCR)

Total RNA was extracted using TRIzol reagent (Thermo Fisher Scientific) and was reverse-transcribed into cDNA via a Reverse Transcription Kit (Invitrogen). RT-qPCR analysis was performed using SYBR Green Premix PCR Master Mix (Roche, Mannheim, Germany) under ABI HT9600 (Applied Biosystems, Foster City, CA, USA). The relative expression level was normalized to GAPDH or U6 and was calculated by 2^−ΔΔCt^ methods.

### CCK-8 proliferation assays

After transfection, PC-3 or VCaP cells (1 × 10^3^) were plated into 96-well plates for 0, 24, 48, 72, and 96 h. CCK-8 solution was added. Microplate reader (Thermo Fisher Scientific) was applied to measure the optical density at a wavelength of 450 nm.

### Colony formation assay

Cells (1 × 10^3^) were plated into 6-well plates at 37 °C with 5% CO_2_. After 14 days, colonies were fixed with 4% paraformaldehyde (Solarbio, Beijing, China) for 10 min, and dyed with crystal violet (Beyotime, Nantong, China) for 5 min. Colonies were counted manually.

### Western bolt

The extracted total protein was subjected to SDS-PAGE (Boster Biological Technology, LA, CA, USA) and transferred to PVDF membranes. After sealing PVDF with milk, the primary antibodies were added for incubation overnight, including anti-STAT5A (ab32043, Abcam, Cambridge, UK), anti-E-cadherin (ab1416, Abcam), anti-N-cadherin (ab18203, Abcam) and anti-GAPDH (ab8245, Abcam). After adding secondary antibody, protein signals were detected by chemiluminescence detection system (GE Healthcare, Chicago, IL, USA).

### Transwell invasion assay

Transfected cells in non-serum medium were seeded into top chamber (Corning, NY, USA) pre-coated with Matrigel (Millipore, MA, USA). The lower inserts were filled with FBS. Fixed cells were dyed with crystal violet (Sigma). Photos were taken under a digital microscope (Nikon, Tokyo, Japan).

### Wound healing assay

Subsequent to transfection, PC cells plated in 6-well plates underwent 4 h serum starvation. Thereafter, the wound was stimulated by making a straight scratch in the cell monolayer with application of the sterile 200 μl pipette tip. After gently rising the scratched monolayer twice using a serum-free medium, the wound was allowed to heal in the complete medium for 24 h. Then, picture of wound width at 0 and 24 h was captured respectively applying an inverted microscope following the wound formation. Migration of PC cells was assessed by examining % wound closure.

### Flow cytometry analysis

After being cultured at 6-well plates for 48 h, cells were fixed in 70% ethanol pre-cooled with ice for 2 h. Quantification of apoptosis was measured by flow cytometry (Thermo Fisher Scientific) after staining with Annexin V-labeled with 7AAD and PE (BD Biosciences, San Jose, CA, USA).

### Luciferase reporter assay

The wild-type plasmids of SNHG17/STAT5A sequences containing the putative binding sites of miR-339-5p and their mutations were cloned into the pmirGLO dual-luciferase vector (Promega, Madison, WI, USA), termed SNHG17 WT/Mut and STAT5A WT/Mut. The plasmids were co-transfected with miR-339-5p mimic and NC mimic into 239T cell with Lipofectamine 2000 (Invitrogen). The promoter sequences of SNHG17, SNORA71B or STAT5A was sub-cloned into pGL3 luciferase vector to construct promoter plasmids, then co-transfected with indicated transfection plasmids into 239T cells. Luciferase activities were tested with Dual-Luciferase Reporter Assay System (Promega).

### Subcellular fractionation

The cytoplasmic and nuclear extracts were extracted from PC-3 cell with Nuclear and Cytoplasmic Extraction Reagents (Thermo Fisher Scientific). RNAs isolated from nucleus or cytoplasm was analyzed with RT–qPCR analysis. The levels of U6 (nucleus control), GAPDH (cytoplasm control) were measured, respectively.

### RNA fluorescence in situ hybridization (FISH)

The RNA FISH probe mix for SNHG17 was synthesized and produced by RiboBio (Guangzhou, China). DAPI from RiboBio was used to counterstain cell nuclei. Cells were observed with FV1000 confocal laser microscope (Olympus, Tokyo, Japan).

### RNA immunoprecipitation (RIP) assay

RIP assays were conducted using Millipore EZ-Magna RIP kit (Millipore, Billerica, MA, USA). Cell lysates were detected with antibody against Ago2 and normal IgG attached with magnetic beads, followed by RT-qPCR.

### DNA pull-down assay

DNA pull-down test kit was used in line with user guide (Gzscbio, Guangzhou, China). Biotin-labeled promoter bound with streptavidin magnetic beads were cultured with cellular protein extracts at 4 °C overnight, and separated by SDS-PAGE, followed by RT-qPCR.

### Immunofluorescence (IF)

Antibodies against E-cadherin and N-cadherin were added at 4 °C overnight. Cells nuclei were counterstained with DAPI. Cells were detected via Olympus Fluoview 1000 confocal microscope.

### Chromatin immunoprecipitation (ChIP) assay

The Magna ChIP Kit (Millipore) was utilized for ChIP assay. Cross-linked chromatin was sonicated to 200–500-bp fragments and immunoprecipitated with antibody prior to RT-qPCR.

### In vivo experiment

Transfected PC-3 cells were injected into BALB/c male nude mice. Twenty-eight days later, mice were killed. Tumor volume and weight were calculated. Animal experiment was approved by the Animal Experiments Ethics Committee of the Jiangxi cancer hospital. Metastasis nodules were observed using HE staining.

### Immunohistochemistry (IHC) assay

Fresh samples were cut to proper size and fixed in 4% paraformaldehyde. Sections were analyzed by IHC using antibodies against Ki67, PCNA, E-cadherin and N-cadherin.

### Statistical analysis

Data of triplicate experiments were analyzed by SPSS (SPSS Inc., Chicago, IL, USA) and GraphPad Prism 5 (GraphPad Software, San Diego, CA). Results were denoted as means ± SD. Correlation of SNHG17 and SNORA71B with PFS in PC patients was assessed by Kaplan–Meier analysis and log-rank test. Significance was evaluated by *t*-test or ANOVA. *P* < 0.05 indicated statistical significance.

## Results

### SNHG17 and SNORA71B were upregulated in PC, and SNHG17 positively regulated SNORA71B

**Fig. 1:**
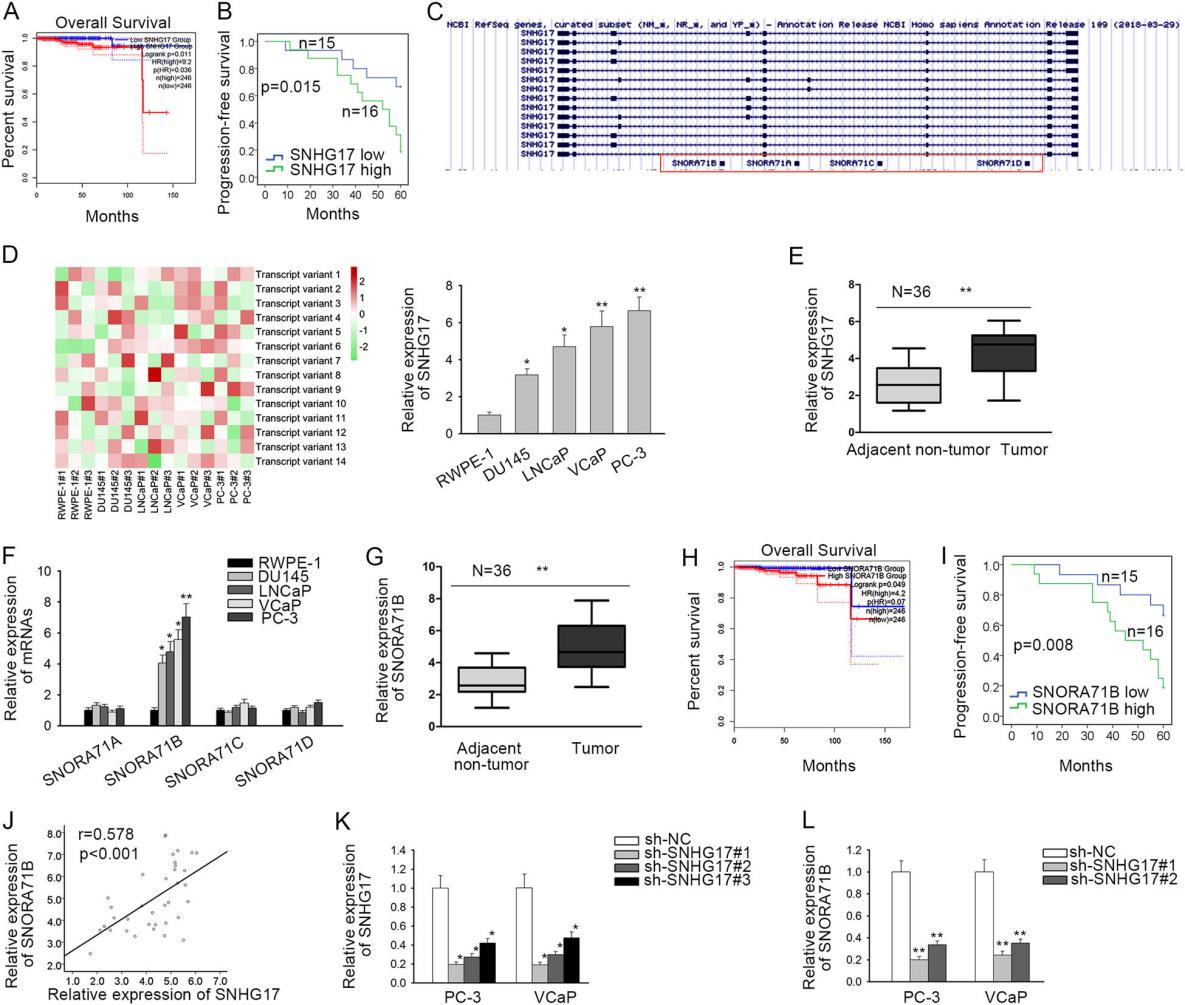
SNHG17 and SNORA71B were upregulated in PC, and SNHG17 positively regulated SNORA71B. **a** GEPIA analysis of TCGA data showed that high expression of SNHG17 indicated poor prognosis of PC. **b** Association of SNHG17 with progression-free survival in PC patients was analyzed by Kaplan–Meier analysis and log rank test. **c** UCSC showed 14 transcript variants of SNHG17. **d** RT-qPCR showed the expression of 14 transcript variants of SNHG17 in PC cell lines and normal cell line. **e** Expression of SNHG17 in PC tissues and matched para-tumorous tissues. **f** Expression of SNORA71A, SNORA71B, SNORA71C, SNORA71D in PC cell lines and normal cell line. **g** Expression of SNORA71B in PC tissues and matched para-tumorous tissues. **h** GEPIA analysis of TCGA data showed that high expression of SNORA71B indicated poor prognosis of PC. **i** Association of SNORA71B with progression-free survival in PC patients was analyzed by Kaplan–Meier analysis and log-rank test. **j** Pearson’s correlation curve showed the positive correlation between SNORA71B and SNHG17 in PC samples. **k** RT-qPCR of the knockdown efficiency of SNHG17 in PC cells. **l** RT-qPCR expression of SNORA71B in PC cells under SNHG17 silence. *P < 0.05, **P < 0.01.

In the beginning, we probed the implication of SNHG17 in PC. TCGA data analysis through GEPIA (http://gepia2.cancer-pku.cn/#index) revealed that high expression of SNHG17 denoted unfavorable prognosis of prostate adenocarcinoma (PRAD) patients (Fig. 1a). Kaplan–Meier analysis depicted that high SNHG17 level indicated low progression-free survival (PFS) in PC patients (Fig. 1b). By UCSC genome browser, SNHG17 was identified to have 14 transcript variants (Fig. 1c). Therefore, primers were designed to distinguish these transcript variants and expression of each transcript was detected in cell lines to find out which transcript of SNHG17 majorly contributed to the expression of SNHG17 in PC. Results showed that transcript variant 6, which was 1034 nt long, was highly expressed in 4 PC cell lines compared with the normal cell line (Fig. 1d), indicating that the high expression of SNHG17 in PC was determined by this 1034 nt transcript. Thereafter, we validated SNHG17 upregulation in PC tissues compared with the paired adjacent non-tumor tissues (Fig. 1e).

Besides, we found that SNHG17 had 4 homolog snoRNAs: SNORA71A, SNORA71B, SNORA17C, and SNORA71D (Fig. 1b). Recent studies have revealed that snoRNAs also participate in the carcinogenesis of various cancers^14^. Considering the homologous relation between SNHG17 and the 4 snoRNAs, we deduced that the snoRNAs might also play a part in PC and SNHG17 potentially regulated these snoRNAs. By detecting the expressions of these snoRNAs in cell lines, we found that among 4 snoRNAs, only SNORA71B was significantly upregulated in PC cell lines versus the normal cell line (Fig. 1f). In addition, elevated level of SNORA71B was confirmed in PC tissues (Fig. 1g). Through analyzing TCGA data by GEPIA, we uncovered the positive relation between high SNORA71B and low survival of PRAD patients (Fig. 1h). Also, SNORA71B upregulation was related to awful PFS in PC patients (Fig. 1i). Expressions of SNHG17 and SNORA71B were positively associated in PC samples as shown in the correlation curve in Fig. 1j. Later, the effect of SNHG17 on SNORA71B was detected. Since we showed that expression of SNHG17 determined by transcript variant 6 was the highest in PC-3 and VCaP cell lines, endogenous expression of SNHG17 was silenced in these two cell lines by three specific sh-RNAs targeting this transcript. RT-qPCR data demonstrated that sh-SNHG17#1/2 knocked down SNHG17 in both cell lines more significantly than sh-SNHG17#3 (Fig. 1k). Hence, the 2 sh-RNAs were applied for subsequent experiments. The level of SNORA71B was downregulated upon the silence of SNHG17 in two PC cell lines (Fig. 1l). Additionally, we confirmed that knocking down SNHG17 reduced SNORA71B level in other PC cell lines including DU145, LNCaP, and that overexpression of SNHG17 induced SNORA71B in RWPE-1 cells (Supplementary Fig. 1a). Moreover, we showed that knocking down SNHG17 reduced SNORA71B in NSCLC, gastric cancer, and colorectal cancer cell lines (A549, MGC-803, and DLD1), in which SNHG17 was reported to exert oncogenic functions by former studies^16,34,35^ (Supplementary Fig. 1b). These data indicated that the regulation of SNHG17 on SNORA71B was not prostate-specific. In collection, SNHG17 and SNORA71B were upregulated in PC, and SNHG17 positively regulated SNORA71B expression.

### Knockdown of SNHG17 abrogated proliferation, improved apoptosis, hindered migration, invasion, and EMT in PC cells

**Fig. 2:**
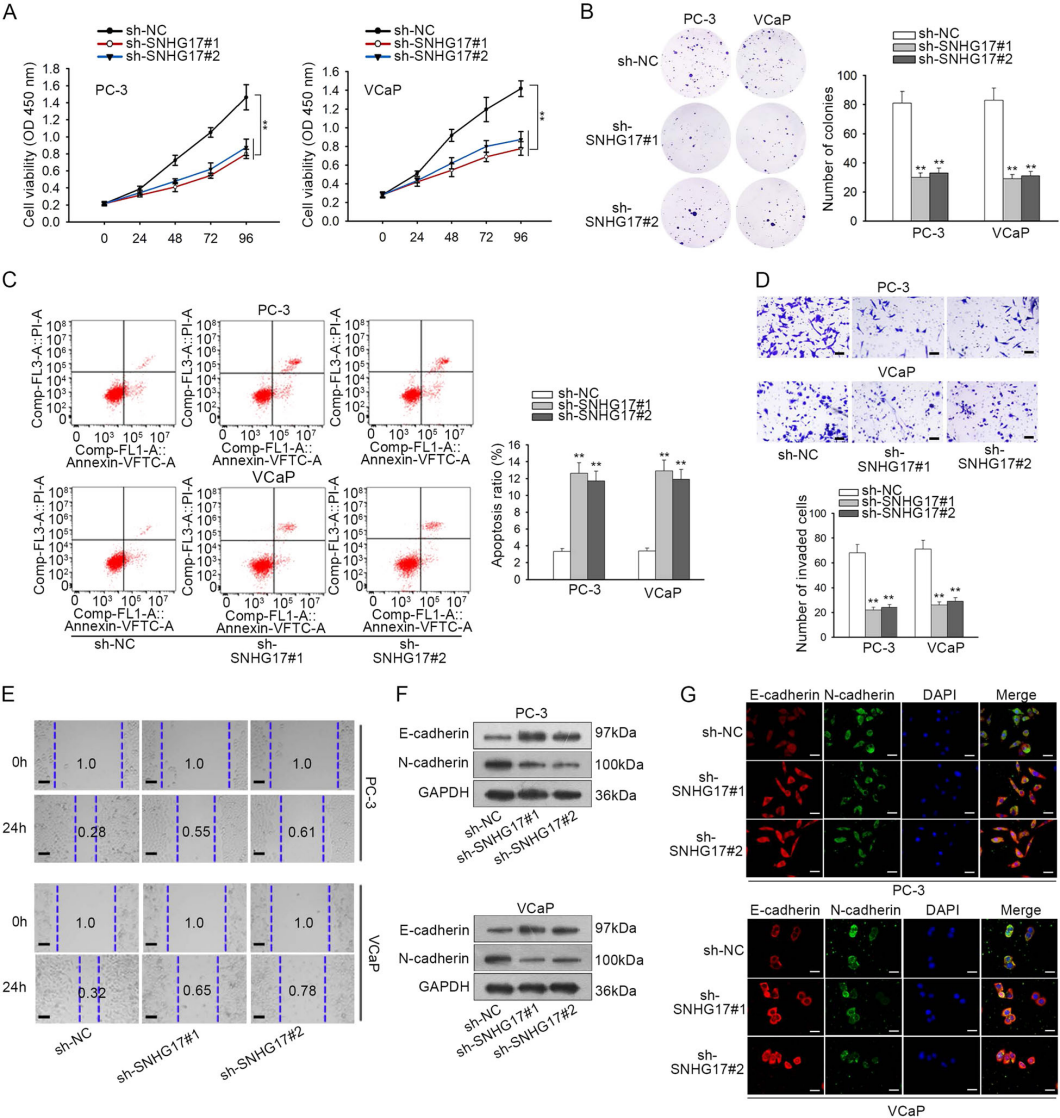
Knockdown of SNHG17 abrogated proliferation, improved apoptosis, hindered invasion and EMT in PC cells. **a**, **b** CCK-8 and colony formation assays were applied to evaluate PC cell proliferation under SNHG17 knockdown. **c** Flow cytometry apoptosis analysis was used to analyze apoptosis of PC cells under SNHG17 silence. **d** Transwell invasion assay was used to examine invasion of PC cells under SNHG17 knockdown. **e** Migration of PC cells was assessed by wound healing assay under SNHG17 silence. **f**, **g** E-cadherin and N-cadherin expressions in PC cells were examined by western blot and IF staining. **P < 0.01.

Next, biological role of SNHG17 in PC cells was determined by a series of loss-of-function assays. Proliferation of PC-3 and VCaP cells was detected by CCK-8 and colony formation assays. Results presented that knockdown of SNHG17 with sh-SNHG17#1/2 reduced the cell viability and colony generation of two PC cell lines, indicating that SNHG17 silence impeded proliferation of PC cells (Fig. 2a, b). Flow cytometry cell apoptosis analysis depicted that silencing SNHG17 increased apoptosis of PC cells (Fig. 2c). Additionally, the invasive and migratory abilities of PC cells were abolished by SNGH17 depletion (Fig. 2d, e). Moreover, EMT progression in PC cells was examined by analyzing the levels of EMT markers. As shown by western blot and IF staining, E-cadherin expression was increased, whereas N-cadherin expression was decreased by the downregulation of SNHG17 in PC cells (Fig. 2f, g). Jointly, silence of SNHG17 abrogated proliferation, invasion, migration, EMT, and strengthened apoptosis in PC cells.

### Knockdown of SNORA71B hindered PC progression

**Fig. 3:**
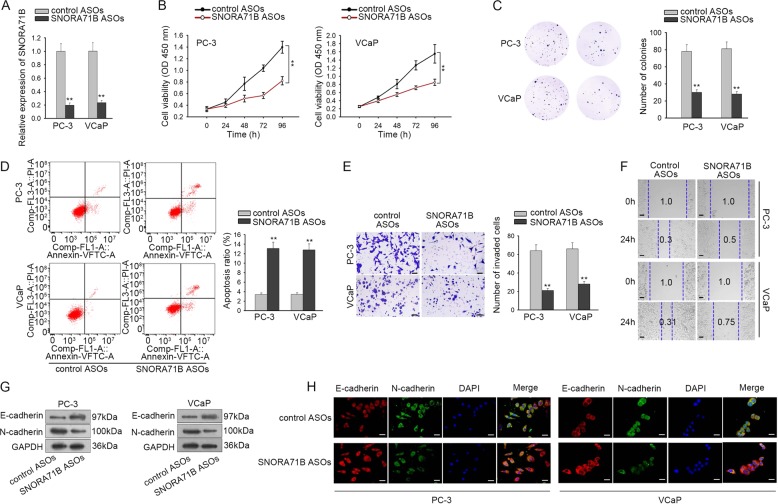
Knockdown of SNORA71B hindered PC progression. **a** RT-qPCR analysis confirmed the knockdown efficiency of SNORA71B in PC cells. **b**, **c** CCK-8 and colony formation assays were applied to evaluate PC cell proliferation under SNORA71B knockdown. **d** Flow cytometry apoptosis analysis was used to analyze apoptosis of PC cells under SNORA71B silence. **e** Transwell invasion assay was used to examine invasion of PC cells under SNORA71B knockdown. **f** Pictures of the width of scratch wounds of PC cells at 0 and 24 h under SNORA71B silence. **g**, **h** E-cadherin and N-cadherin expressions in PC cells were examined by western blot and IF staining. **P < 0.01.

Meanwhile, impact of SNORA71B on PC progression was tested. SNORA71B was silenced by specific ASOs in PC-3 and VCaP cells. RT-qPCR data verified the overt downregulation of SNORA71B in two PC cell lines by the transfection of SNORA71B ASOs compared with the control ASOs (Fig. 3a). Similarly, we observed that depletion of SNORA71B prohibited proliferation, facilitated apoptosis, retarded invasion, migration, and EMT in PC cells (Fig. 3b–h). Hence, it was validated that SNORA71B silence inhibited PC progression.

### STAT5A transcriptionally induced SNHG17 and SNORA71B

**Fig. 4:**
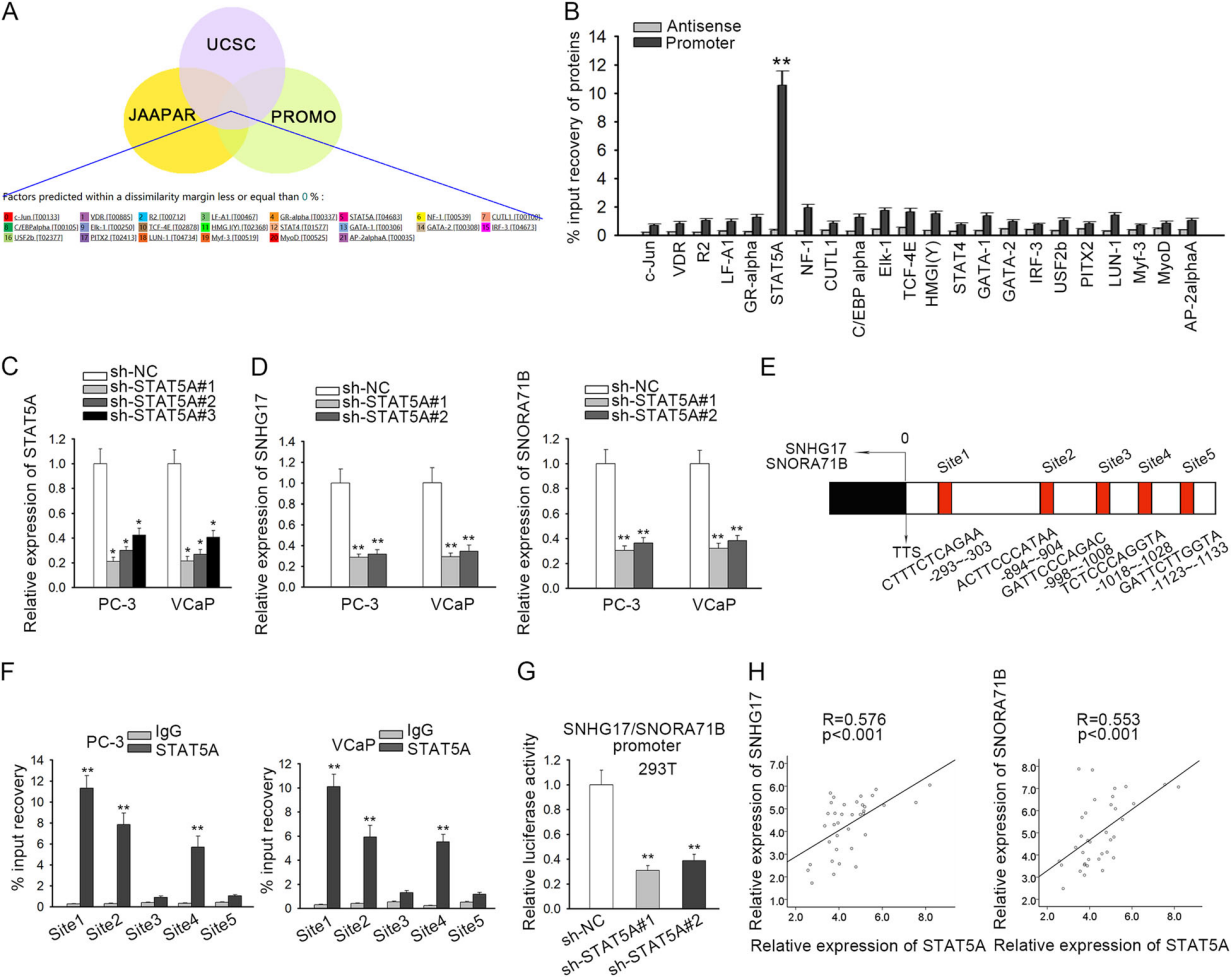
STAT5A transcriptionally induced SNHG17 and SNORA71B. **a** Venn pattern showed 22 TFs for SNHG17 and SNORA71B promoter predicted by JASPAR, UCSC, and PROMO. **b** Pulldown assay showed the interaction of STAT5A with SNHG17 and SNORA71B promoter. **c** RT-qPCR analysis confirmed the knockdown efficiency of STAT5A in PC cells. **d** RT-qPCR expression of SNHG17 and SNORA71B in PC cells under SNHG17 silence. **e** 5 STAT5A sites on SNHG17 and SNORA71B promoter were predicted by JASPAR. **f** ChIP assay showed that STAT5A bound to site 1, 2, and 4 of SNHG17 and SNORA71B promoter. **g** Luciferase reporter assay was used to evaluate the effect of STAT5A on transcription of SNHG17 and SNORA71B promoter. **h** Pearson’s correlation curve showed the positive correlation of STAT5A with SNORA71B and SNHG17 in PC samples. *P < 0.05, **P < 0.01.

Then, we tried to explain the mechanism of SNHG17 and SNORA71B upregulation in PC cells. Since SNHG17 and SNORA71B were spliced from the exons and introns respectively from the same primary RNA transcript, it is theoretically recognized that SNHG17 and SNORA71B shared the same promoter. As widely reported, the dynamic binding of transcription factors (TFs) to the degenerate DNA motifs on the promoter leads to the activation of certain oncogenes in cancer cells^36^. Hence, we speculated that SNHG17 and SNORA71B might be activated by TFs in PC cells. With the application of three prediction tools: JASPAR (http://jaspar.genereg.net/), PROMO (http://alggen.lsi.upc.es/cgi-bin/promo_v3/promo/promoinit.cgi?dirDB=TF_8.3), and UCSC (http://genome.ucsc.edu/), we identified 22 TFs potentially bound to the promoter of SNHG17 and SNORA71B (Fig. 4a). To figure out their interactions with the promoter, pulldown assay was applied. Results revealed that STAT5A was significantly enriched in the pulldown of SNHG17/SNORA71B promoter rather than the antisense promoter (Fig. 4b). STAT5A has been identified as a transcriptional activator which is upregulated in PC and contributed to the progression of PC^37–39^. Therefore, we speculated that STAT5A was mainly responsible for the transactivation of SNHG17 and SNORA71B.

To detect the impact of STAT5A on SNHG17 and SNORA71B, we silenced STAT5A in PC cells (Fig. 4c). Consequently, we observed that knockdown of STAT5A downregulated SNHG17 and SNORA71B in PC cells (Fig. 4d). Furthermore, we identified 5 potential binding sites for STAT5A at the promoter region through JASPAR tool (Fig. 4e). ChIP analysis confirmed that the DNA fragments containing site 1, 2, and 4 were enriched in the immunoprecipitation products of STAT5A (Fig. 4f), indicating that STAT5A bound to the promoter of SNHG17 and SNORA71B promoter at site 1, 2, and 4. Luciferase reporter assay showed that silence of STAT5A led to the decrease of luciferase activity of promoter reporter (Fig. 4g). Besides, we confirmed that STAT5A was positively correlated to SNHG17 and SNORA71B in PC samples (Fig. 4h). Altogether, the aforementioned data indicated that STAT5A transcriptionally induced SNHG17 and SNORA71B.

### SNHG17/miR-339-5p/STAT5A positive feedback loop positively regulated SNORA71B expression

**Fig. 5:**
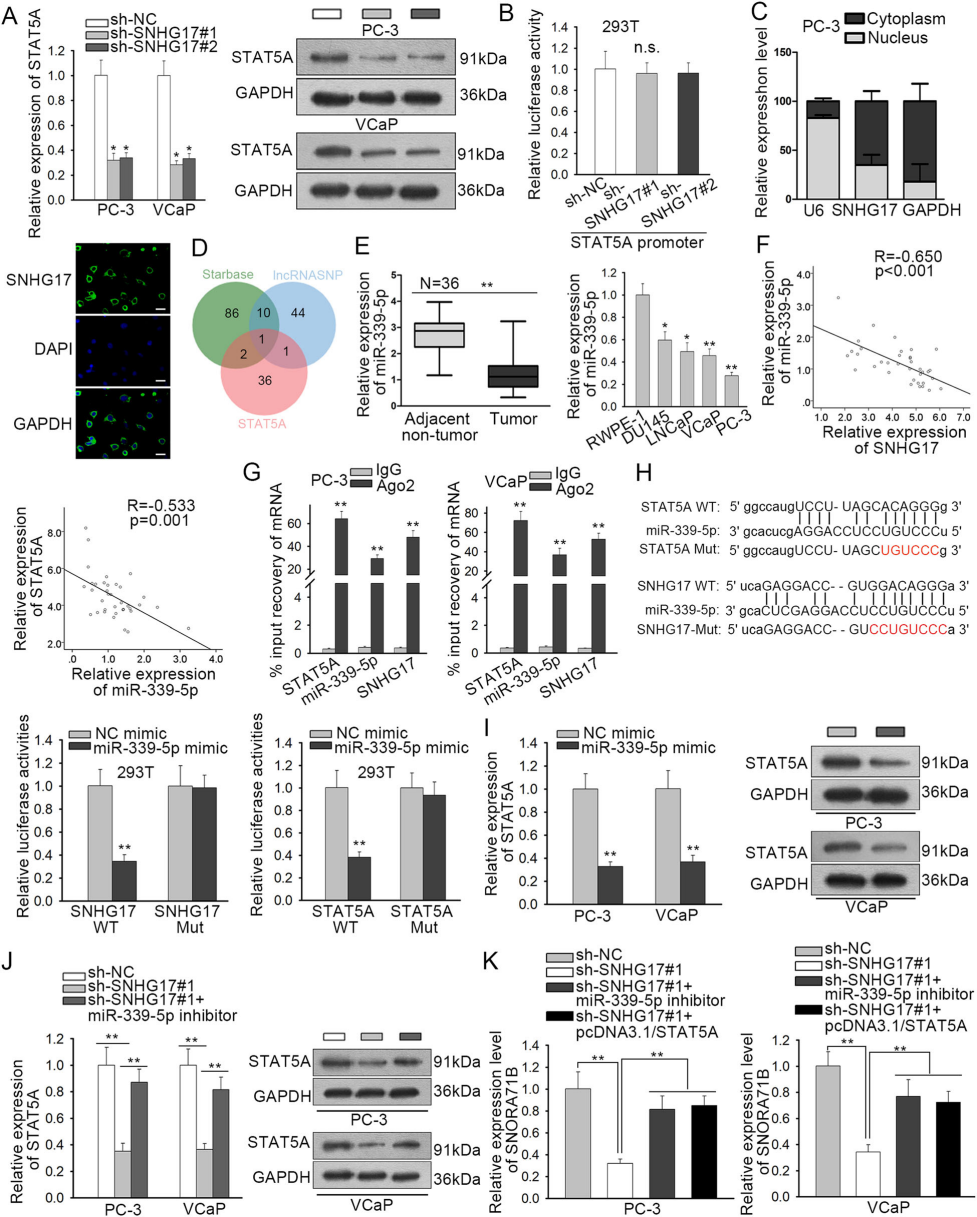
SNHG17/miR-339-5p/STAT5A positive feedback loop positively regulated SNORA71B expression. **a** RT-qPCR and western blot assay showed the expression of STAT5A level under SNHG17 silence in PC cells. **b** Luciferase reporter assay showed that SNHG17 cannot regulate transcription of STAT5A. **c** Subcellular localization of SNHG17 in PC cells was determined by subcellular fractionation and FISH. **d** Venn pattern showed that Starbase and lncRNASNP predicted miR-339-5p as shared miRNA for SNHG17 and STAT5A. **e** Expression of miR-339-5p in PC tissues and cell lines was detected by RT-qPCR. **f** Pearson’s correlation curve showed the negative correlation of miR-339-5p with STAT5A and SNHG17 in PC samples. **g** RIP assay confirmed the interaction of miR-339-5p with STAT5A and SNHG17 in PC cells. **h** Binding sites of miR-339-5p on STAT5A and SNHG17 were obtained from Starbase. Luciferase reporter assay confirmed the interaction of miR-339-5p with STAT5A and SNHG17. **i** RT-qPCR and western blot assays showed the expression of STAT5A level under miR-339-5p overexpression in PC cells. **j** RT-qPCR and western blot assays showed the expression of STAT5A level under indicated transfection in PC cells. **k** RT-qPCR results of SNORA71B expression under indicated transfection in PC cells. *P < 0.05, **P < 0.01.

Subsequently, we explored the mechanism behind the regulation of SNHG17 on SNORA71B expression. Since we have confirmed that STAT5A activated SNORA71B transcriptionally, we wondered whether SNHG17 could regulate the expression of SNORA71B through STAT5A. We found that silence of SNHG17 reduced STAT5A mRNA and protein expressions (Fig. 5a). However, silence of SNHG17 failed to affect the transcription activity of STAT5A (Fig. 5b), indicating that SNHG17 potentially regulated STAT5A at post-transcriptional level. Then, subcellular fractionation and FISH staining revealed the cytoplasmic localization of SNHG17 expression in PC-3 cells (Fig. 5c). Large quantities of reports demonstrated that lncRNAs post-transcriptionally modulate the downstream genes through sequestering miRNAs^10^. Therefore, we sought to find out the miRNAs through which SNHG17 regulated STAT5A in PC. We predicted the target miRNAs for SNHG17, especially for transcript variant 6, through Starbase3.0 (http://starbase.sysu.edu.cn/) and lncRNASNP (http://bioinfo.life.hust.edu.cn/lncRNASNP#!/lncrna_info?lncrna=NONHSAT079660.2), and predicted the miRNAs targeting STAT5A through Starbase3.0. As presented by the Venn pattern in Fig. 5d, only miR-339-5p was in the intersection of the prediction results. Several studies have revealed that miR-339-5p was a tumor-repressing gene in multiple cancers, such as ovarian cancer^30^, lung adenocarcinoma^31^, breast cancer^32^, and colorectal cancer^33^. Thus, we deduced that miR-339-5p was involved in the regulation of SNHG17 on STAT5A in PC. RT-qPCR analysis demonstrated that miR-339-5p was downregulated in PC samples and cell lines (Fig. 5e). As shown by the correlation curve, miR-339-5p level was negatively associated with SNHG17 and STAT5A level in PC samples (Fig. 5f).

Then, we interrogated the interaction of miR-339-5p with SNHG17 and STAT5A. Through RIP analysis, miR-339-5p was co-immunoprecipitated with SNHG17 and STAT5A mRNA by Ago2 (Fig. 5g), indicating that miR-339-5p could interact with SNHG17 or STAT5A in an RNA-induced silencing complex (RISC). Besides, the miR-339-5p binding sequences on SNHG17 and STAT5A were obtained from Starbase3.0 and presented in Fig. 5h, together with the mutated sites. The overexpression of miR-339-5p inhibited luciferase activity of SNHG17 WT and STAT5A WT, whereas SNHG17 Mut and STAT5A Mut exhibited no significant alteration (Fig. 5h). In subsequence, we detected the regulation of miR-339-5p on STAT5A. Overexpression of miR-339-5p decreased STAT5A mRNA and protein levels in PC cells (Fig. 5i). Moreover, inhibiting miR-339-5p reversed the inhibitory effect of SNHG17 silence on STAT5A expression at mRNA and protein levels (Fig. 5j). Finally, we tried to detect whether SNHG17 regulated SNORA71B expression through miR-339-5p/STAT5A axis. We observed that miR-339-5p inhibitor or pcDNA3.1/STAT5A restored SNORA7B expression that was reduced by SNHG17 silence in PC cells (Fig. 5k). In a word, the data above implied that SNHG17/miR-339-3p/STAT5A positive feedback loop positively regulated SNORA71B expression in PC.

### SNHG17 drove PC progression through SNORA71B

**Fig. 6:**
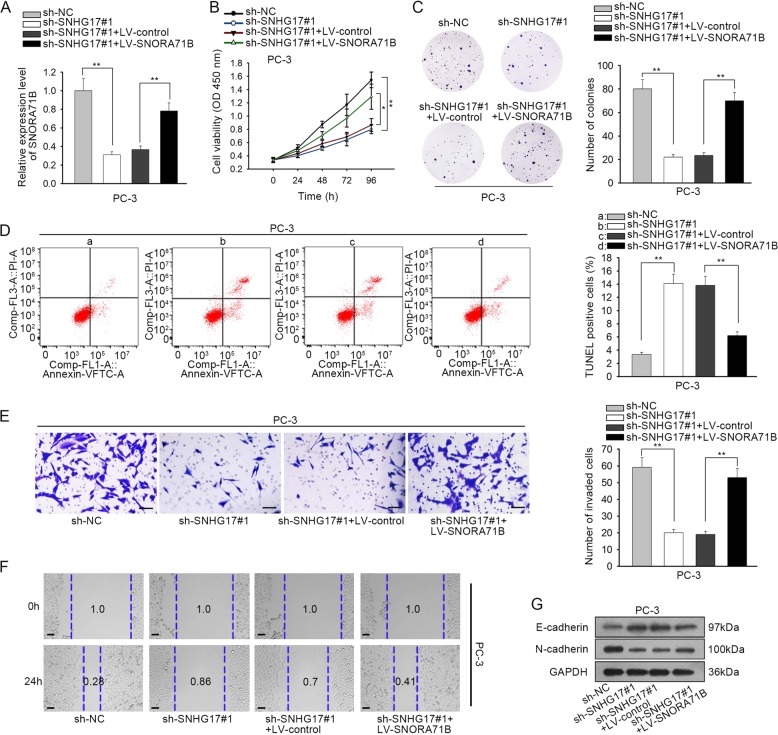
SNHG17 drove PC progression through SNORA71B. **a** RT-qPCR results of SNORA71B expression under indicated transfection in PC cells. **b**, **c** CCK-8 and colony formation assays were applied to evaluate PC cell proliferation under indicated transfection in PC cells. **d** Flow cytometry apoptosis analysis was used to analyze apoptosis of PC cells under indicated transfection in PC cells. **e**, **f** Transwell invasion assay and wound healing assay were used to examine invasion and migration of PC cells under indicated transfection in PC cells. **g** E-cadherin and N-cadherin expressions in PC cells were examined by western blot. *P < 0.05, **P < 0.01.

To investigate whether SNHG17 regulated PC progression through SNORA71B, we designed rescue assays. It was confirmed that knockdown of SNHG17 reduced the level of SNORA71B in PC-3 cells, and co-transfection of LV-SNORA71B restored the expression of SNORA71B (Fig. 6a). The inhibitory impact of sh-SNHG17#1 on PC-3 cell proliferation was abolished by the overexpression of SNORA71B (Fig. 6b, c). Increased apoptosis of PC-3 cells under SNHG17 silence could be counteracted by the overexpression of SNORA71B (Fig. 6d). SNORA71B overexpression recovered the invasion and migration of PC-3 cells with SNHG17 silence (Fig. 6e, f). The increase of E-cadherin and decrease of N-cadherin in SNHG17-silenced PC-3 cells was reversed by SNORA71B overexpression (Fig. 6g). In collection, SNHG17 drove PC progression through a SNORA71B-required way.

### SNHG17 facilitated PC tumor growth and metastasis in vivo

**Fig. 7:**
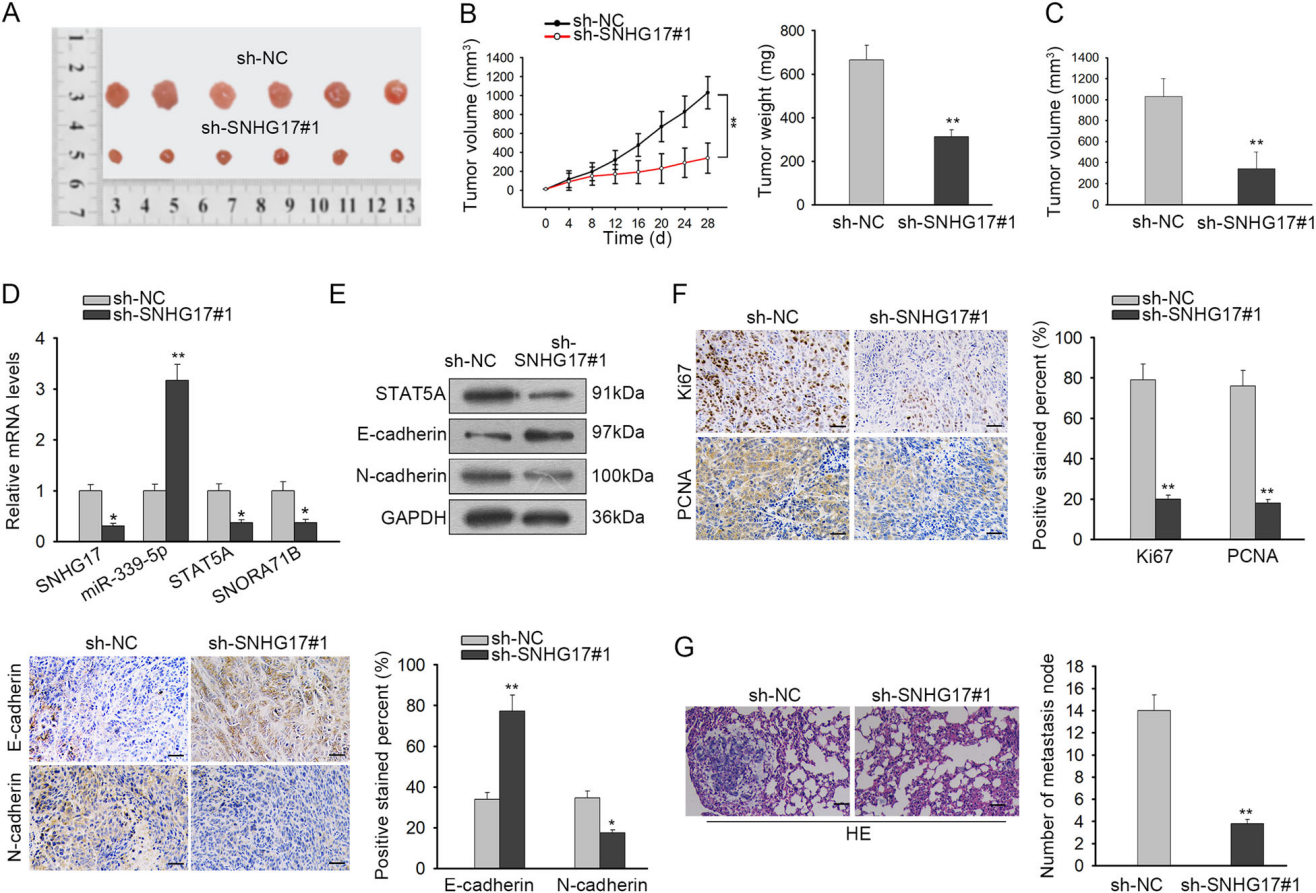
SNHG17 facilitated PC tumor growth and metastasis in vivo. **a** Pictures of xenografts of mice injected with PC-3 cells transfected with sh-NC or sh-SNHG17#1 after being resected. **b** Growth curve and final tumor weight of xenografts from mice in each group. **c** Final volume of xenografts of mice in each group. **d** Expression of SNHG17, miR-339-5p, STAT5A, and SNORA71B of xenografts of mice in each group was tested by RT-qPCR. **e** Western blot results of STAT5A, E-cadherin, and N-cadherin in xenografts of mice in each group. **f** IHC staining of Ki67, PCNA, E-cadherin, and N-cadherin in xenografts of mice in each group. **g** HE staining of metastatic nodes in xenografts of mice in each group. **P < 0.01.

Finally, we detected the function of SNHG17 in PC in vivo. PC-3 cells transfected with sh-NC or sh-SNHG17#1 were injected into nude mice to generate xenografts. As a result, silence of SNHG17 led to the generation of smaller PC tumors in mice (Fig. 7a, Supplementary Fig. 1c). The growth curve showed that SHNG17 knockdown retarded PC tumor growth in vivo (Fig. 7b). The tumor weight and volume were reduced by the knockdown of SNHG17 (Fig. 7b, c). Also, we confirmed that silence of SNHG17 reduced the expression of SNHG17, STAT5A, and SNORA71B levels, and induced miR-339-5p levels in xenografts (Fig. 7d). Western blot results showed that STAT5A and N-cadherin levels were decreased and E-cadherin level was increased by the SNHG17 depletion in xenografts of mice (Fig. 7e). Also, IHC staining showed that the proliferation marker Ki67 and PCNA showed a lower positivity in the SNHG17-silenced tumors from mice, and that the stain positivity of E-cadherin was increased whereas N-cadherin decreased in the SNHG17-silenced tumors from mice (Fig. 7f). Moreover, HE staining showed that SNHG17 reduced the metastatic nodules in SNHG17-silenced tumors from mice (Fig. 7g). In conclusion, SNHG17 facilitated PC tumor growth and metastasis in vivo.

## Discussion

The prostate cancer development involves multiple factors such as mRNA splicing, DNA methylation, and dysregulation of steroidogenic enzyme caused by androgen stimulation^2^. Aberrant expression of lncRNAs in carcinogenesis and progression of PC has been unceasingly revealed during the past decades. Notably, lncRNAs transcribed from SNHGs are reported to be promising biomarkers for the diagnosis and prognosis of human cancers including PC^11–13^. Herein, we discovered through GEPIA bioinformatics analysis that high SNHG17 level in PRAD patients indicated unfavorable prognosis and confirmed that high SNHG17 expression denoted poor PFS, indicating that SNHG17 might be related to PC. Previously, several works demonstrated the high expression and oncogenic properties of SNHG17 in cancers such as gastric cancer and colorectal cancer^16,17^. Recent study also explained that the upregulation of SNHG17 in NSCLC was related to copy number^34^. Herein, we found that SNHG17 had 14 transcript variants through UCSC, and revealed that transcript variant 6 which is 1034 nt in length was the major contributor of the high expression of SNHG17 in PC cell lines and tissues. However, whether the upregulation of this transcript was correlated to copy number will be explored in the future.

Interestingly, it is known that SNHG-transcribed lncRNAs are spliced from the exons of host gene, whereas their homolog snoRNAs are spliced from the introns^14^, which suggested that lncRNA SNHGs potentially correlated with the homolog snoRNAs. Also, a recent study showed that SNHG10 positively regulated its homolog snoRNA SCARNA13 to promote hepatocarcinogenesis^40^. In our study, we found that among four homolog snoRNAs for SNHG17, only SNORA71B was highly expressed in PC cell lines and samples and associated with dismal outcomes of PC patients according to TCGA data. Moreover, we uncovered that SNHG17 positively regulated and correlated with SNORA71B in PC. Besides, we validated that SNHG17 positively regulated SNORA71B in all PC cell lines we applied and also in other cancer cell lines in which SNHG17 was identified as an oncogene^16,34,35^. Functionally, we showed that silencing either SNHG17 or SNORA71B abolished proliferation, migration, invasion, and EMT, whereas induced apoptosis in PC cells. These findings indicated that SNHG17 and SNORA71B were positively correlated and functioned as oncogenes in PC.

Accumulating evidence have proved that transcriptional activation of lncRNAs is a major cause of their upregulation in cancer cells^41,42^. TFs are therefore recognized as crucial regulators of the dysregulation of lncRNAs at transcription level. Herein, we revealed that STAT5A was the major contributor of the transcription of SNHG17 and SNORA71B through pulldown assay. STAT5A is one of a homologous isoform of STAT5, which acts as a TF to regulate cellular response to growth factors and cytokines^37^. STAT5A can be phosphorylated and activated typically by Jak2, forming the STAT5A/B dimers, translocated to the nucleus, and interacting with DNA^37^. High expression of STAT5A and its homolog STAT5B has been revealed in PC^38^. Also, STAT5A has been proved to drive the cell proliferation and tumor growth in PC^39^. In accordance, we validated that STAT5A bound to the shared promoter of SNHG17 and SNORA71B to induce their transcription and expressions.

Volumes of studies have depicted the ceRNA mechanism through which lncRNAs regulate their target genes. Herein, we tried to investigate whether SNHG17 regulated STAT5A to influence SNORA71B expression. Bioinformatics tool revealed that miR-339-5p interacted with both STAT5A and SNHG17. Formerly, miR-339-5p was identified to hinder the development of multiple cancers through impairing cell proliferation and metastasis^30–33^. We firstly proved that miR-339-5p was downregulated in PC and negatively correlated with SNHG17 and STAT5A. Furthermore, we validated the interaction of miR-339-5p with SNHG17 and STAT5A. In addition, we confirmed that SNHG17 upregulated STAT5A through miR-339-5p and therefore induced SNORA71B. Rescue assays confirmed that SNHG17 facilitated PC progression through miR-339-5p and SNORA71B, which indicated the role of SNHG17/miR-339-5p/STAT5A/SNORA71B axis in PC. Finally, animal experiments validated that SNHG17 drove tumor growth and metastasis in vivo.

**Fig. 8:**
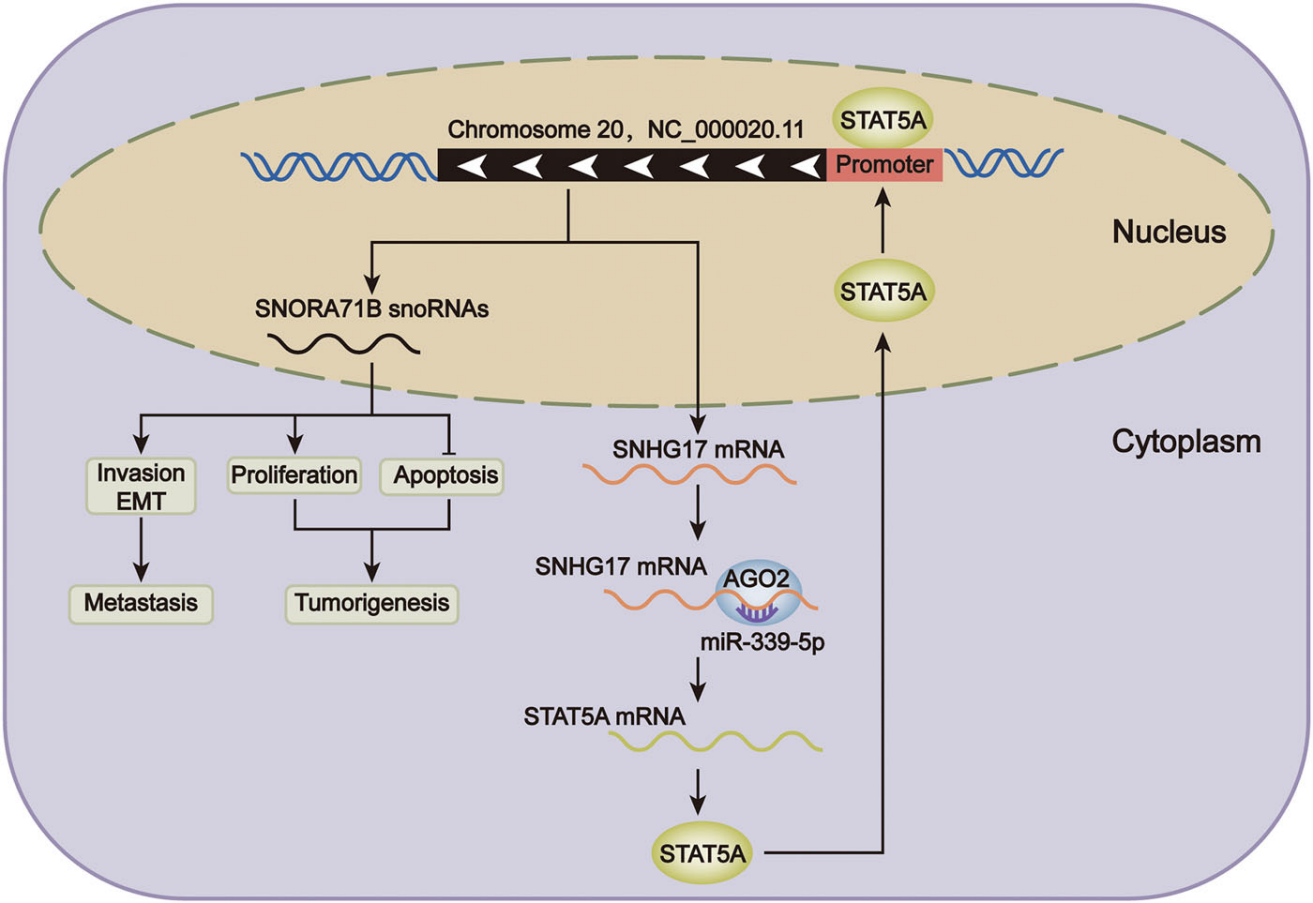
Graphical abstract. SNHG17/miR-339-5p/STAT5A positive feedback loop induced the expression of SNORA71B to facilitate proliferation, impede apoptosis, and promote invasion and EMT in vitro, and drove tumorigenesis and metastasis of PC in vivo.

In conclusion, we firstly demonstrated that SNHG17 and its homolog SNORA71B were oncogenic genes in PC. Functionally, SNHG17 and SNORA71B promoted proliferation, migration, invasion, and EMT, whereas hindered apoptosis in vitro, and SNHG17 drove tumor growth in vivo. Mechanistically, we revealed that SNHG17 and SNORA71B were transcriptionally activated by STAT5A. Furthermore, SNHG17/miR-339-5p/STAT5A positive feedback loop induced the expression of SNORA71B in PC cells (Fig. 8). These findings potentially provides new thoughts to pursue a higher treatment efficacy and better prognosis in PC patients.

## Supplementary information

Supplementary figure legend

Supplementary figure 1

## References

[1] Attard G (2016). Prostate cancer. Lancet.

[2] Bostwick DG (2004). Human prostate cancer risk factors. Cancer.

[3] Bonkhoff H, Berges R (2010). From pathogenesis to prevention of castration resistant prostate cancer. Prostate.

[4] Cheetham SW, Gruhl F, Mattick JS, Dinger ME (2013). Long noncoding RNAs and the genetics of cancer. Br. J. Cancer.

[5] Cai Weiliang, Wu Bowen, Li Zhizhong, He Peiheng, Wang Biao, Cai Anlie, Zhang Xiping (2018). LncRNA NBR2 inhibits epithelial‐mesenchymal transition by regulating Notch1 signaling in osteosarcoma cells. Journal of Cellular Biochemistry.

[6] Chen M (2019). LINC01939 inhibits the metastasis of gastric cancer by acting as a molecular sponge of miR-17-5p to regulate EGR2 expression. Cell Death Dis..

[7] Cheng R, Li N, Yang S, Liu L, Han S (2018). Long non-coding RNA ZEB1-AS1 promotes cell invasion and epithelial to mesenchymal transition through inducing ZEB1 expression in cervical cancer. OncoTargets Ther..

[8] Cimadamore A (2017). Long non-coding RNAs in prostate cancer with emphasis on second chromosome locus associated with prostate-1 expression. Front. Oncol..

[9] Zhu M (2014). lncRNA H19/miR-675 axis represses prostate cancer metastasis by targeting TGFBI. FEBS J..

[10] Li Jianping, Zhang Zhipeng, Xiong Li, Guo Chuan, Jiang Tao, Zeng Lilan, Li Ge, Wang Juan (2017). SNHG1 lncRNA negatively regulates miR-199a-3p to enhance CDK7 expression and promote cell proliferation in prostate cancer. Biochemical and Biophysical Research Communications.

[11] Huang W (2017). The long non-coding RNA SNHG3 functions as a competing endogenous RNA to promote malignant development of colorectal cancer. Oncol. Rep..

[12] Wu Jie, Zhao Wei, Wang Zhonghou, Xiang Xu, Zhang Shengchi, Liu Lina (2018). Long non-coding RNA SNHG20 promotes the tumorigenesis of oral squamous cell carcinoma via targeting miR-197/LIN28 axis. Journal of Cellular and Molecular Medicine.

[13] Lv P (2018). Long non-coding RNA SNHG6 enhances cell proliferation, migration and invasion by regulating miR-26a-5p/MAPK6 in breast cancer. Biomed. Pharmacother..

[14] Williams GT, Farzaneh F (2012). Are snoRNAs and snoRNA host genes new players in cancer?. Nat. Rev. Cancer.

[15] Weinstein LB, Steitz JA (1999). Guided tours: from precursor snoRNA to functional snoRNP. Curr. Opin. Cell Biol..

[16] Zhang G (2019). LncRNA SNHG17 promotes gastric cancer progression by epigenetically silencing of p15 and p57. J. Cell. Physiol..

[17] Ma Zhonghua, Gu Shengying, Song Min, Yan Changsheng, Hui Bingqing, Ji Hao, Wang Jirong, Zhang Jianping, Wang Keming, Zhao Qinghong (2017). Long non-coding RNA SNHG17 is an unfavourable prognostic factor and promotes cell proliferation by epigenetically silencing P57 in colorectal cancer. Molecular BioSystems.

[18] Schulten H-J (2017). Comprehensive molecular biomarker identification in breast cancer brain metastases. J. Transl. Med..

[19] Bartel DP (2009). MicroRNAs: target recognition and regulatory functions. Cell.

[20] Bartel DP (2004). MicroRNAs: genomics, biogenesis, mechanism, and function. Cell.

[21] Chen Y-L, Xu Q-P, Guo F, Guan W-H (2017). MicroRNA-302d downregulates TGFBR2 expression and promotes hepatocellular carcinoma growth and invasion. Exp. Therapeutic Med..

[22] Chu P, Liang A, Jiang A, Zong L (2018). miR-205 regulates the proliferation and invasion of ovarian cancer cells via suppressing PTEN/SMAD4 expression. Oncol. Lett..

[23] Hu X (2018). miRNA-103a-3p promotes human gastric cancer cell proliferation by targeting and suppressing ATF7 in vitro. Mol. Cells.

[24] Fang L-L (2017). Potent inhibition of miR-34b on migration and invasion in metastatic prostate cancer cells by regulating the TGF-β pathway. Int. J. Mol. Sci..

[25] Hatano K (2015). A functional screen identifies miRNAs that inhibit DNA repair and sensitize prostate cancer cells to ionizing radiation. Nucleic Acids Res..

[26] Josson S, Sung S-Y, Lao K, Chung LWK, Johnstone PAS (2008). Radiation modulation of microRNA in prostate cancer cell lines. Prostate.

[27] Chan Jia, Tay Yvonne (2018). Noncoding RNA:RNA Regulatory Networks in Cancer. International Journal of Molecular Sciences.

[28] Zhao CC (2019). Lnc SMAD5-AS1 as ceRNA inhibit proliferation of diffuse large B cell lymphoma via Wnt/β-catenin pathway by sponging miR-135b-5p to elevate expression of APC. Cell Death Dis..

[29] Zhao L, Liu B (2017). Identification of potential prognostic ceRNA module biomarkers in patients with pancreatic adenocarcinoma. Oncotarget.

[30] Shan W, Li J, Bai Y, Lu X (2016). miR-339-5p inhibits migration and invasion in ovarian cancer cell lines by targeting NACC1 and BCL6. Tumor Biol..

[31] Li P (2018). miR-339-5p inhibits lung adenocarcinoma invasion and migration by directly targeting BCL6. Oncol. Lett..

[32] Yan H (2016). Prolactin inhibits BCL6 expression in breast cancer cells through a microRNA-339-5p-dependent pathway. J. Breast Cancer.

[33] Zhou C, Lu Y, Li X (2015). miR-339-3p inhibits proliferation and metastasis of colorectal cancer. Oncol. Lett..

[34] Xu T (2019). Gene amplification-driven long noncoding RNA SNHG17 regulates cell proliferation and migration in human non-small-cell lung cancer. Mol. Ther. Nucleic Acids.

[35] Ma Zhonghua, Gu Shengying, Song Min, Yan Changsheng, Hui Bingqing, Ji Hao, Wang Jirong, Zhang Jianping, Wang Keming, Zhao Qinghong (2017). Long non-coding RNA SNHG17 is an unfavourable prognostic factor and promotes cell proliferation by epigenetically silencing P57 in colorectal cancer. Molecular BioSystems.

[36] Wong ES (2015). Decoupling of evolutionary changes in transcription factor binding and gene expression in mammals. Genome Res..

[37] Levy DE, Darnell JE (2002). STATs: transcriptional control and biological impact. Nat. Rev. Mol. Cell Biol..

[38] Li H (2004). Activation of signal transducer and activator of transcription 5 in human prostate cancer is associated with high histological grade. Cancer Res..

[39] Dagvadorj A, Kirken RA, Leiby B, Karras J, Nevalainen MT (2008). Transcription factor signal transducer and activator of transcription 5 promotes growth of human prostate cancer cells *in vivo*. Clin. Cancer Res..

[40] Lan T (2019). LncRNA SNHG10 facilitates hepatocarcinogenesis and metastasis by modulating its homolog SCARNA13 via a positive feedback loop. Cancer Res..

[41] Chen J-F (2018). STAT3-induced lncRNA HAGLROS overexpression contributes to the malignant progression of gastric cancer cells via mTOR signal-mediated inhibition of autophagy. Mol. Cancer.

[42] Chen X (2018). SP1-induced lncRNA-ZFAS1 contributes to colorectal cancer progression via the miR-150-5p/VEGFA axis. Cell Death Dis..

