# Supplementary material for: LncRNA SNHG17 aggravated prostate cancer progression through regulating its homolog SNORA71B via a positive feedback loop
Source: Cell Death Dis. 2020 May 23;11(5):393. doi: 10.1038/s41419-020-2569-y (PMC7245601; doi:10.1038/s41419-020-2569-y)
Supplement: Supplementary file 1 — Supplementary figure legend [file 41419_2020_2569_MOESM1_ESM.docx]

**Supplementary figure 1 Regulation of SNHG17 on SNORA71B in PC cell lines and other cancer cell lines.** (A-B) All cell lines except RWPE-1 were transfected with sh-NC or sh-SNHG17#1/2. RWPE-1 cells were transfected with pcDNA3.1 or pcDNA3.1/SNHG17. RT-qPCR data showed the expression of SNORE71B in all cell lines with indicated transfection compared with sh-NC or pcDNA3.1 control. (C) Pictures of mice transplanted with xenografts derived from PC-3 cells transfected with sh-NC or sh-SNHG17#1 before tumor resection. ^*^P < 0.05, ^**^P < 0.01.
