# Supplementary figures and images for: LncRNA SNHG17 aggravated prostate cancer progression through regulating its homolog SNORA71B via a positive feedback loop
Source: Cell Death Dis. 2020 May 23;11(5):393. doi: 10.1038/s41419-020-2569-y (PMC7245601; doi:10.1038/s41419-020-2569-y)

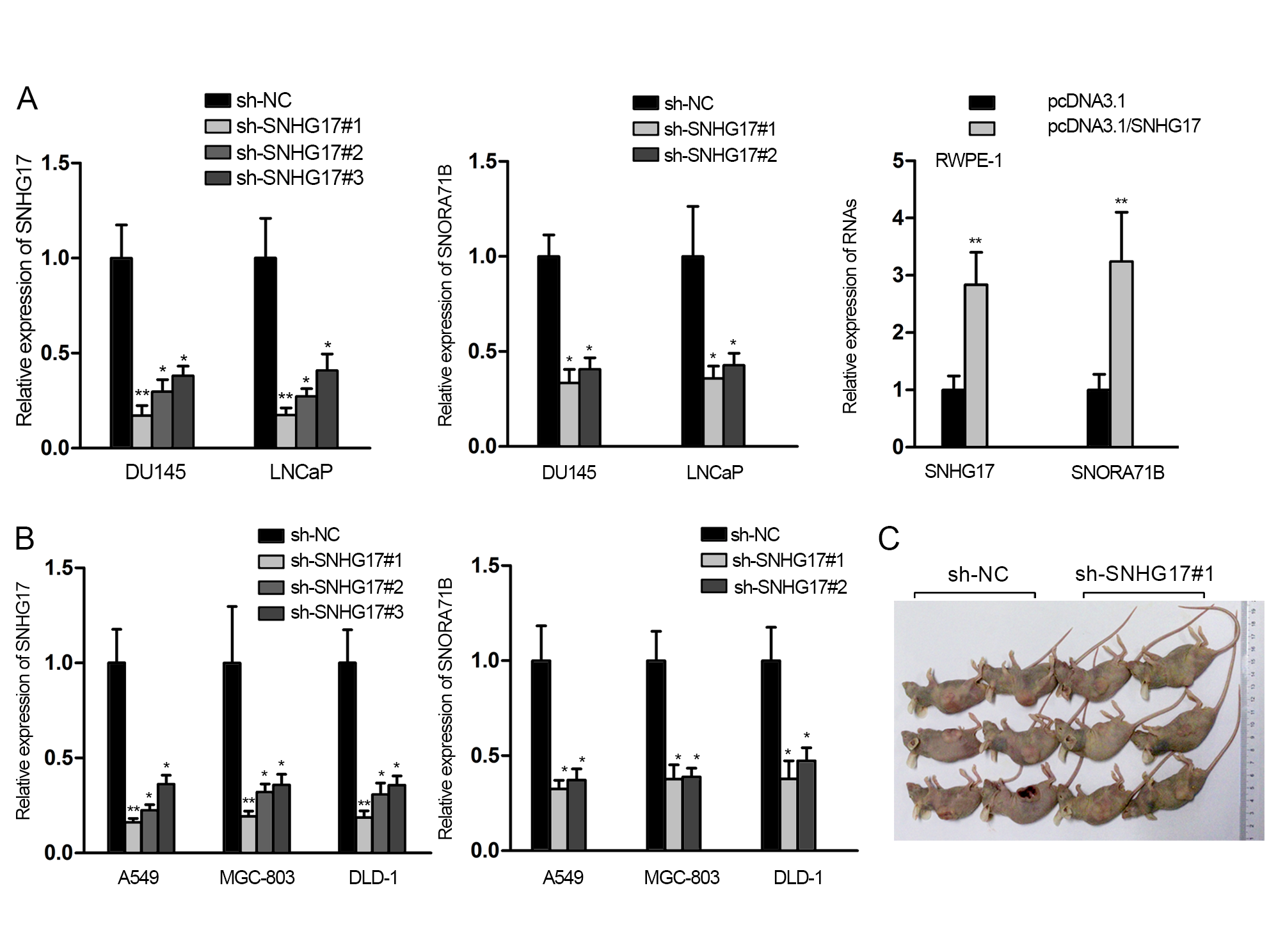

Supplement: Supplementary file 2 — Supplementary figure 1 [file 41419_2020_2569_MOESM2_ESM.tif]
